# The impact of green low-carbon development on public health: a quasi-natural experimental study of low-carbon pilot cities in China

**DOI:** 10.3389/fpubh.2024.1470592

**Published:** 2024-10-08

**Authors:** Zhanjie Wang, Xinyue Wang, Zhichao Wang, Sheng Mai

**Affiliations:** ^1^School of Business Administration, Guizhou University of Finance and Economics, Guiyang, China; ^2^Institute of Gui-An New District, Guizhou University of Finance and Economics, Guiyang, China; ^3^School of Economy & Management, Shihezi University, Shihezi, China

**Keywords:** green low-carbon development, public health, climate change, environmental pollution, low-carbon pilot cities

## Abstract

**Background:**

In recent years, climate change and environmental pollution have posed significant threats to public health. As environmental policies such as low-carbon city initiatives are progressively implemented, their role in enhancing public health has become a topic of growing interest. This study aimed to investigate the relationship between green low-carbon development and public health and to analyze the underlying mechanisms.

**Methods:**

We utilized data from 271 prefecture-level cities in China spanning from 2007 to 2020, focusing on green low-carbon development, climate change, environmental pollution, and public health. Employing the quasi-natural experimental framework of China’s low-carbon city pilot projects, we constructed a multi-site difference-in-differences (DID) model for empirical analysis. Various robustness checks, including parallel trend tests, placebo tests, sample selection bias checks, and adjustments to the temporal and spatial scope of the samples, were conducted to ensure the reliability of the results. Additionally, we explored the positive effects of green low-carbon development on public health through dual mediation pathways involving climate change mitigation and pollution reduction. Finally, we examined the heterogeneity of the results across different city tiers, economic growth rates, levels of technological investment, and green finance development.

**Results:**

The findings indicate that green low-carbon development significantly enhances public health, a conclusion supported by robustness tests. Mechanism analysis reveals that the benefits of green low-carbon development on public health are realized through mitigating climate change and reducing environmental pollution. Further analysis reveals that the positive impact on public health is more pronounced in first-and second-tier cities, as well as in cities with faster economic growth, greater technological investment, and more developed green finance sectors.

**Discussion:**

This study highlights the crucial role of urban green low-carbon development in improving environmental quality and public health. In addition to providing empirical evidence that supports the promotion of green low-carbon development in cities, the results point to policy recommendations for enhancing public health. Moreover, the findings contribute to the development of environmental policies and the implementation of the “Healthy China” strategy.

## 1 Introduction

Since the implementation of the reform and opening-up policies, China’s economy has rapidly developed, making it the second-largest economy globally. However, this sustained economic growth has also resulted in significant ecological damage and environmental pollution (1). Air pollution issues that took a century to develop in Western countries have emerged in concentrated regions in China within just 40 years of economic development. Notably, smog pollution events, primarily caused by PM2.5 and PM10, have become frequent, with an expanding pollution range and increasing severity (2). The “2023 China Ecological Environment Bulletin” reports that out of 339 monitored cities at the prefecture level and above, 136 still fail to meet PM2.5 standards, with the proportion of heavily polluted days increasing by 1.4 percentage points compared to 2022.^1^ This prevalent smog pollution not only endangers public health and well-being but also threatens the construction of an ecological civilization and green low-carbon growth (3). To address these issues and improve public health, the Chinese government has enacted various policies and regulations. Among these, low-carbon city construction stands out as a crucial pilot initiative, essential for fostering harmonious coexistence between humans and nature and advancing the “Healthy China” strategy.

Low-carbon city construction aims to reshape urban areas, utilizing low-carbon thinking and technologies to transform urban production and lifestyles. The goal is to minimize greenhouse gas emissions, thereby fostering a healthy, simple, and low-carbon way of life and consumption model, ultimately achieving inclusive green growth (4, 5). In pursuing the low-carbon development strategy, the Chinese government has designated cities as core areas for implementation, gradually advancing through low-carbon city pilots to meet the “dual carbon goals” of peaking carbon emissions by 2030 and achieving carbon neutrality by 2060. Cities are pivotal in this effort, as they account for approximately three-quarters of global energy consumption (6). Moreover, cities possess abundant resources and diverse tools crucial for addressing climate change challenges (7). While existing research mainly focuses on the effects of low-carbon city pilots on carbon emission reductions (8), pollution control (9), and achieving economic and environmental benefits (10), their impact on public health has been largely overlooked. Can green low-carbon development enhance public health as anticipated? What are the underlying mechanisms? Is there heterogeneity in its effects?

To address these questions, this study leverages the quasi-natural experimental scenario of low-carbon city pilots, using annual data from Chinese cities at the prefecture level and above from 2007 to 2020. We constructed a multi-period DID model to evaluate the impact of green low-carbon development on public health, providing theoretical references for promoting ecological civilization construction and enhancing national health literacy. The robustness tests, including parallel trend tests and placebo tests, yielded consistent results, increasing the credibility and scientific validity of the research findings. Additionally, the mediation mechanism analysis indicated that green low-carbon development affects public health by mitigating climate change and improving environmental quality, clarifying the pathways through which green low-carbon development promotes public health. Furthermore, considering the differences in spatial scale, resource endowment, technological innovation, and financial openness among various cities, this paper further explores the heterogeneity of the impacts from four aspects: city level, economic growth, technological investment, and green finance. This provides valuable insights for examining the differential effects and causes of green low-carbon development on public health and enhancing the driving forces of green low-carbon development.

The study’s marginal contributions are as follows: First, in terms of the research topic, unlike existing studies that emphasize the economic outcomes of low-carbon city construction (8, 9), this paper focuses on the impact of low-carbon city pilot policies on public health, exploring the proactive response of the government during policy adjustments and providing a new perspective for achieving the “Healthy China” strategy. Second, regarding data collection, this study collected data from 271 sample cities between 2007 and 2020, including the complete lists of the first, second, and third batches of low-carbon pilot cities, resulting in 3,466 sets of sample data. This method overcomes the limitations of qualitative analysis (11) and case studies (12), providing a more comprehensive understanding of the value logic and evolutionary dynamics of low-carbon pilot cities. Additionally, it improves the generalizability and applicability of the research findings, offering empirical evidence to support the continued establishment of low-carbon pilot cities nationwide. Third, from a theoretical perspective, green low-carbon development positively impacts public health by addressing climate change and enhancing environmental quality. This helps to elucidate the mechanisms through which green low-carbon development benefits public health, further advancing research on the impact of atmospheric environmental policies and pollution on public health (13, 14). Finally, for practical significance, we examine the heterogeneity of impacts while considering factors such as city tier, economic growth, technological investment, and green finance, revealing the health distribution effects of city pilot policies. This study provides empirical insights for developing diversified low-carbon city pilot programs, improving policy combinations, and establishing a multi-actor coordinated public health governance system.

**Figure 1:**
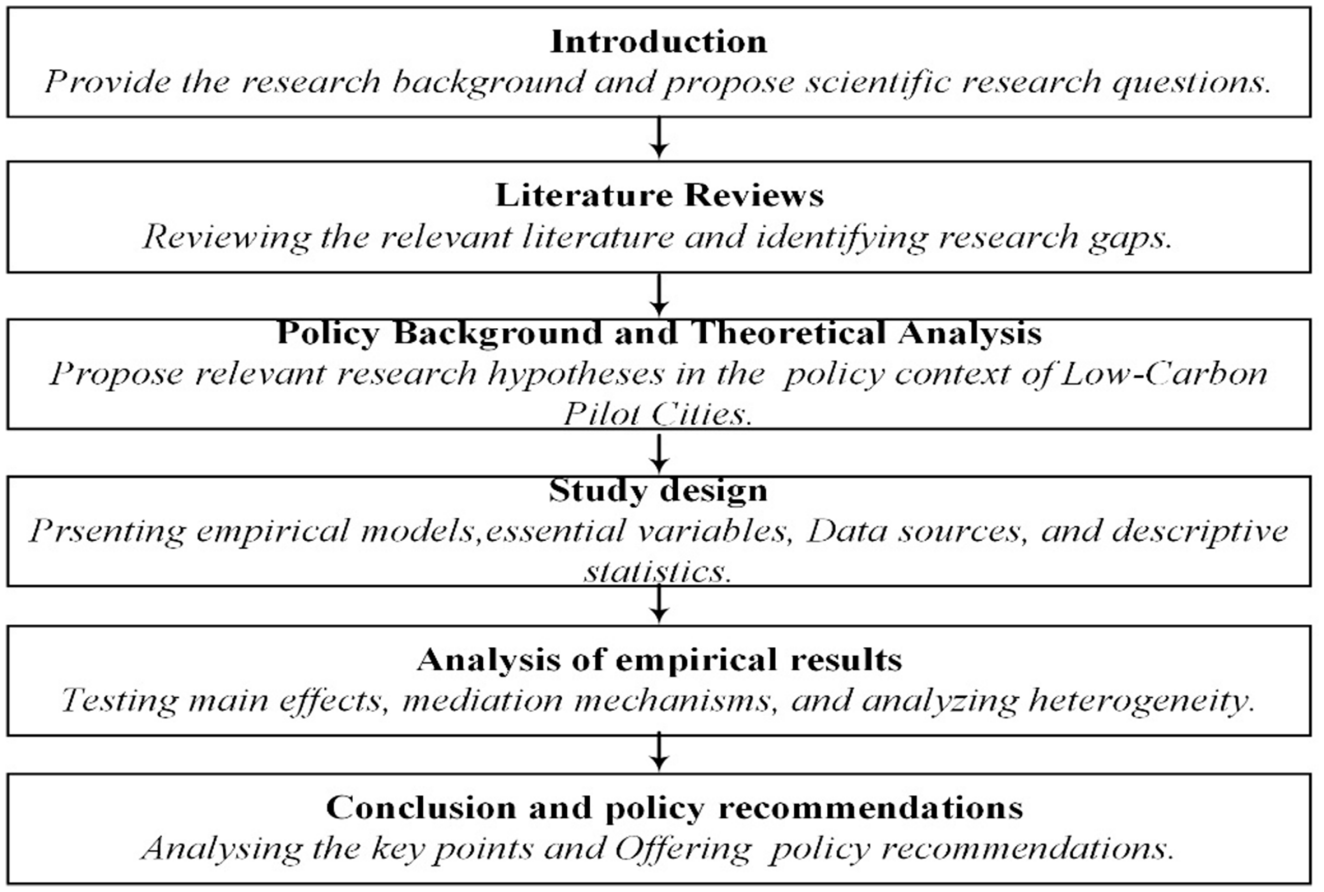
Research framework graph.

The remainder of this paper is organized as follows: Section 2 reviews the literature on green low-carbon development and public health. Section 3 introduces the policy background of low-carbon pilot cities and proposes hypotheses. Section 4 describes the models, variables, and data sources. Section 5 examines the influence of green low-carbon development on public health empirically, conducted a series of robustness tests and analyzed heterogeneity. Section 6 summarizes our conclusions and policy implications, and presents research limitations and future prospects. The specific research design is shown in Figure 1.

## 2 Literature review

**Figure 2:**
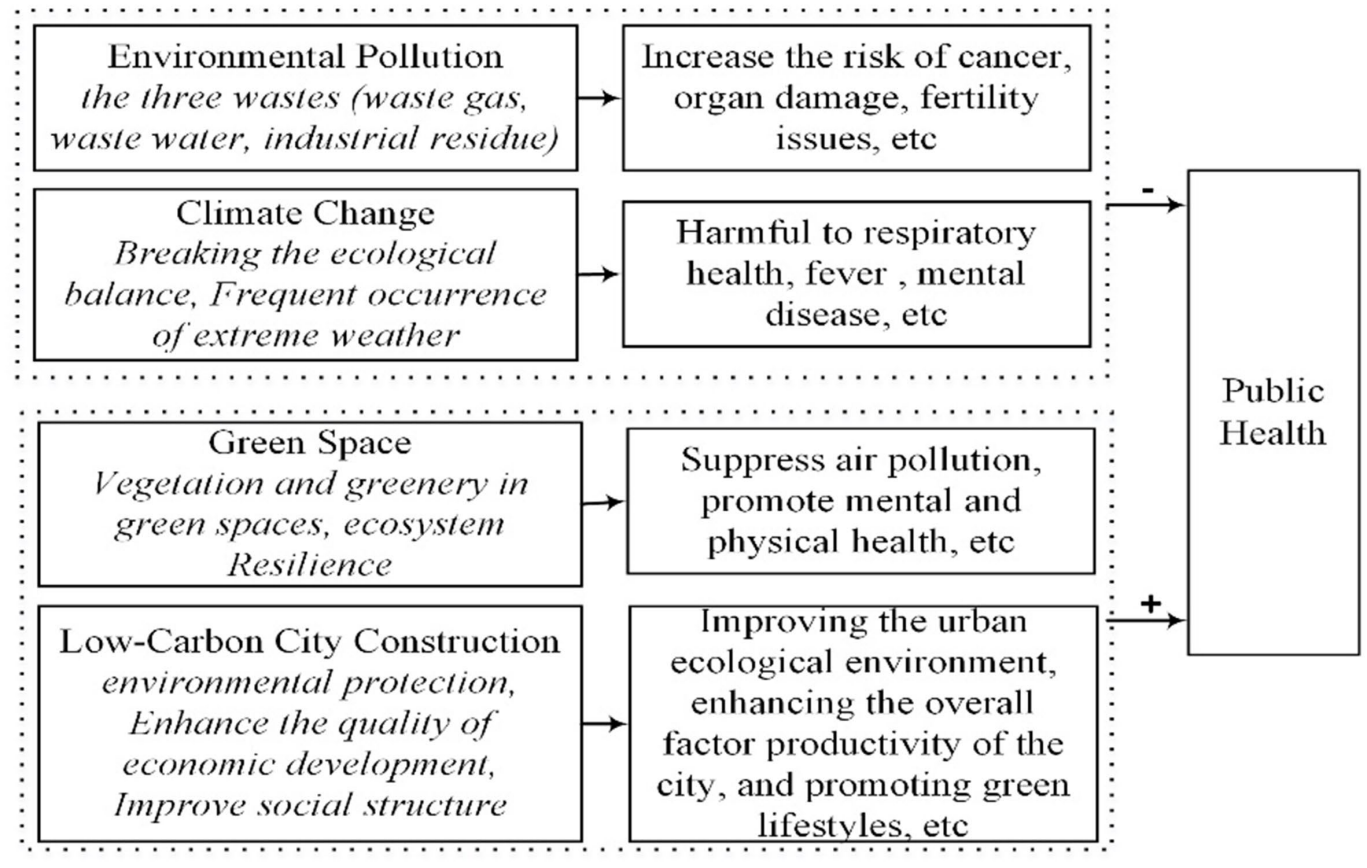
Literature review flowchart.

The natural environment, as a space for social activities, is closely linked to public health. Scholars have explored the relationship between environment and public health, primarily focusing on environmental pollution, climate change, and green spaces (Figure 2). Environmental pollution has become one of the leading environmental factors causing disease and premature deaths worldwide (14, 15). Especially during urbanization, the discharge of industrial waste gases, wastewater, and solid waste leads to severe air, water, and soil pollution, further harming public health. Particulate matter in air pollutants can enter the human body via the respiratory tract, negatively affecting the respiratory, cardiovascular, and central nervous systems and increasing cancer risk (16, 17). Water pollution impacts groundwater quality, and long-term exposure to contaminated water can cause organ damage, developmental issues, and reproductive problems (18, 19). Soil pollution by heavy metals accumulates over time, posing significant public health risks when these contaminants enter the food chain (20). In addition to pollution, the extensive use of fossil fuels has led to a sharp increase in carbon dioxide emissions, causing severe climate change. This disrupts the ecological balance and increases the frequency of extreme weather events such as heatwaves, droughts, and wildfires. These events directly impact health. For example, heatwaves can increase local mortality rates (21), and wildfire smoke can harm respiratory health (22). Additionally, such extreme weather can exacerbate existing mental health issues or trigger new psychological disorders (23). What’s more, the impact of environmental factors on public health varies, with pollution and climate change having more significant effects on vulnerable groups and low-to middle-income countries (24). These impacts also extend to social and economic areas, as diseases or deaths caused by pollution and climate change result in substantial economic losses, including medical costs, healthcare costs, and productivity losses due to decreased health and premature death among laborers (25). Moreover, climate change and environmental pollution disproportionately affect low-to middle-income countries and impoverished populations, exacerbating social inequality. On the other hand, green spaces positively affect human health. Vegetation in green spaces helps reduce air pollution (26), and access to these areas promotes both mental and physical health by providing opportunities to connect with nature (27).

Low-carbon cities, as a crucial approach to addressing environmental and climate challenges and achieving sustainable development, offer multiple environmental, economic, and social benefits (Figure 2). Environmentally, low-carbon cities improve energy efficiency, promote green energy transitions (28), reduce urban carbon emissions (29), lower smog pollution (30), and enhance urban ecological environments (31). Economically, they boost cities’ overall technological innovation capacity (32), improve labor allocation in enterprises, optimize labor structures (33), increase industrial capacity utilization rates (34), and enhance total factor productivity (35). Socially, they improve residents’ living environments, raise green awareness (36), guide shifts toward greener lifestyles (37), promote social equity, reduce the wage gap between corporate executives and employees (38), encourage rural–urban migration, and reduce the urban–rural income gap (39). Low-carbon cities represent an update in technology, administrative management, and development models, as well as social structures (40). Their construction requires proper government policy guidance and close cooperation among the government, enterprises, and the public (41). Regarding the policy diffusion effect of low-carbon city pilots, it shows an overall upward trend over time, though issues like stagnation, bias, and variability persist. Spatially, policy promotion exhibits clear proximity effects, ripple effects, and hierarchical characteristics (42).

Research on low-carbon green development and public health has produced significant results, offering methodological support for this study. However, existing studies have focused mainly on the pathological mechanisms of how environmental pollution impacts public health (17, 43), lacking a clear logical pathway from environmental policy to environmental pollution, climate change, and public health. There is a need to clarify how low-carbon city construction can reduce environmental pollution and its subsequent impact on public health. Public health is a key indicator of national prosperity and strength, underscoring the importance of examining the validity of this logical pathway. Furthermore, empirical studies on low-carbon development employ diverse measurement methods, resulting in varying conclusions (34, 38). Finally, many previous studies analyzing the effects of low-carbon green development on public health overlook the issue of endogeneity in influencing factors by relying solely on panel data for empirical analysis (28, 42).

To address the limitations of previous research and mitigate model endogeneity issues, we have implemented the following enhancements. Firstly, we developed a dual mediation model within a multi-scale evaluation theoretical framework for public health. This model enables a comprehensive assessment of the impact of environmental pollution, climate change, and other factors on public health, as well as a thorough exploration of the relationship between green low-carbon development and public health, revealing their interaction pathways. Secondly, we utilized a quasi-natural experiment involving low-carbon city construction pilots and using a DID model to study the effects of low-carbon city construction on public health. By rigorously testing mechanisms and heterogeneity, we were able to partially address endogeneity issues stemming from omitted variables. Lastly, low-carbon city construction serves as a comprehensive environmental regulation tool incorporating market-based incentives and command-and-control measures. This approach helps avoid the limitations of singular and partial environmental regulation indicators, enhancing the persuasiveness of our conclusions.

## 3 Policy background and theoretical analysis

**Figure 3:**
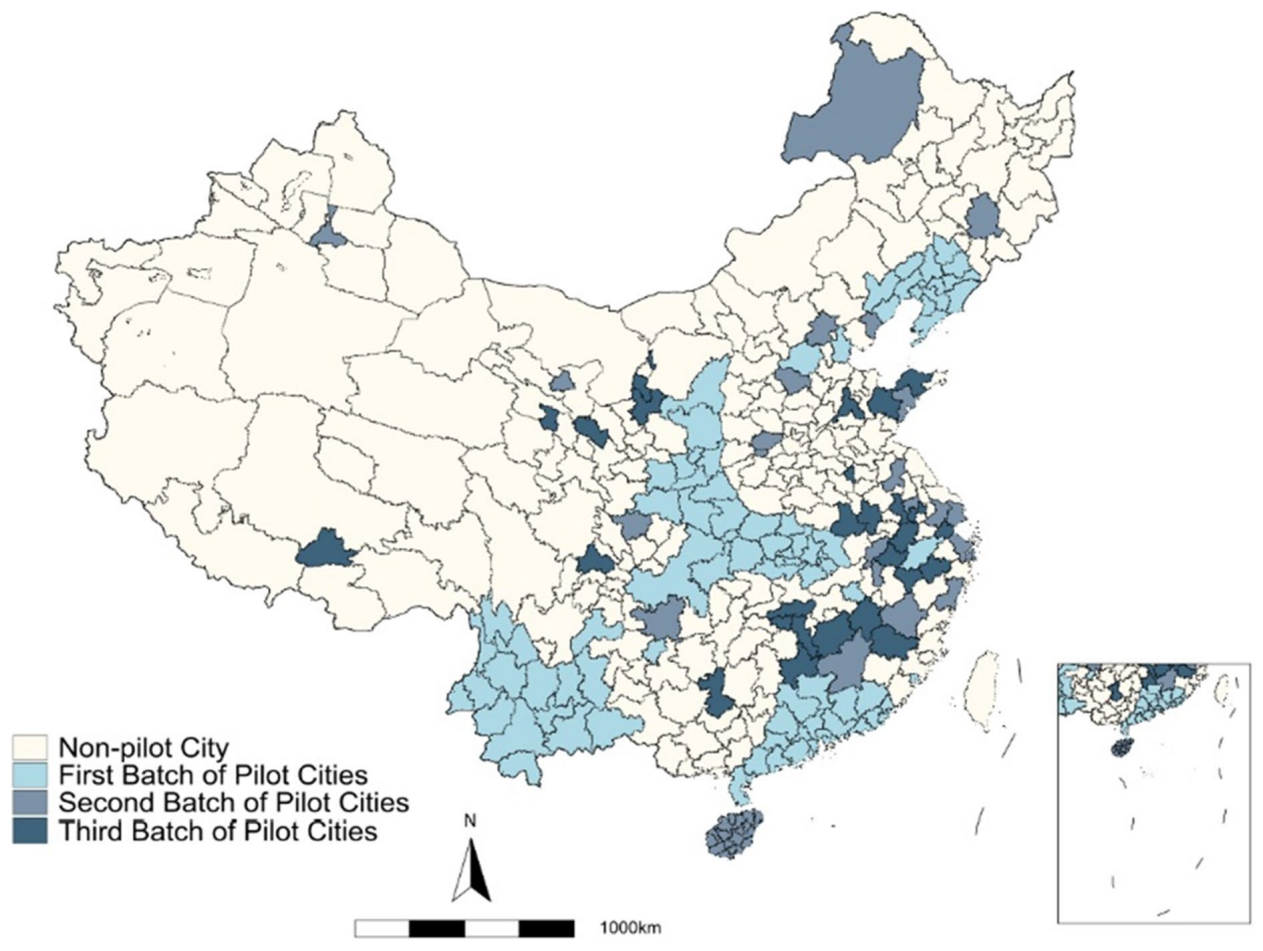
Distribution map of low-carbon pilot cities in China.

To effectively address climate change and environmental pollution, the Chinese government has initiated low-carbon city pilot projects to explore pathways for green socioeconomic development. In July 2010, the National Development and Reform Commission issued a notice announcing the first batch of low-carbon pilots, comprising five provinces and eight prefecture-level cities.^2^ The second and third batches were announced in 2012 and 2017^3^, respectively, bringing the total number of low-carbon city pilots to 81, as shown in Figure 3. These pilot projects aim to develop cities based on a low-carbon economy, with citizens adopting low-carbon lifestyles and the government using low-carbon principles to guide urban construction. The main focus areas include developing low-carbon development plans, creating policies to promote low-carbon industries, establishing a greenhouse gas emission data management system, setting targets for controlling greenhouse gas emissions, and promoting green low-carbon lifestyles and consumption patterns. According to the “2023 National Low-Carbon City Pilot Evaluation Report,” these initiatives have achieved positive outcomes and provided valuable insights for local green development. The low-carbon city pilots also offer a quasi-experimental scenario for studying their impact on public health.

This paper posits that low-carbon city pilot projects can improve public health by mitigating climate change and environmental pollution. These projects use a combination of policy guidance and economic incentives to drive technological advancements, optimize energy structures, adjust economic structures, and enhance carbon sequestration. For instance, by imposing fees and taxes, they increase the costs of pollution and carbon emissions for enterprises, encouraging technological innovation and process improvements (4, 9). Simultaneously, preferential policies like fiscal subsidies and tax reductions lower the costs of technological upgrades, promoting further research and dissemination of clean technologies (44). Additionally, low-carbon city pilots actively promote clean energy sources such as solar and wind power, diversify energy types, optimize energy structures, and reduce reliance on high-carbon, high-pollution energy sources like coal and oil (34, 37). They also slow down the development of high-carbon industries, prioritize low-carbon industries, and raise entry thresholds for heavy industries to fundamentally reduce emissions. For example, in the construction sector, energy-saving residences adhering to low-carbon standards are promoted to achieve zero emissions (29). In the transportation sector, public transportation systems are developed and expanded to reduce reliance on private vehicles, and new energy vehicles are gradually popularized (36). In the consumer sector, the promotion of low-carbon lifestyles enhances residents’ green awareness and encourages low-carbon behaviors. Additionally, low-carbon city pilots increase urban green spaces, improve urban green coverage, and enhance urban carbon sequestration levels (41).

Climate change and environmental pollution impact public health through various channels. Excessive carbon emissions lead to global climate change, resulting in frequent extreme weather events such as heatwaves, wildfires, and floods, which directly affect human health. For example, heatwaves can cause heat exhaustion, heat syncope, and heatstroke, and the surge in patients due to extreme heat events can overwhelm public health systems, impacting the care of other patients (21, 22, 45). Floods can lead to drowning incidents and, in their aftermath, increase the incidence of infectious and parasitic diseases (46), as well as negatively affect public mental health (23). Moreover, these extreme weather events damage infrastructure and buildings, causing significant economic losses and affecting public health expenditure in cities, thereby impacting public health services (47). Low-carbon city pilots help reduce carbon emissions, mitigating the health impacts of extreme weather caused by climate change. Environmental pollution can be categorized into air, water, and soil pollution. Air pollution mainly results from sulfur oxides, nitrogen oxides, PM2.5, and other toxic substances. For instance, PM2.5 can be inhaled into the body due to its small size, pass through the respiratory barriers, enter the circulatory system, and spread throughout the body (48), damaging the respiratory and cardiovascular systems and exacerbating diabetes (49). Similarly, water and soil pollution negatively affect public health. Polluted water bodies and soils often contain toxic substances like heavy metals, organic pollutants, and acidic or alkaline substances. These toxic substances accumulate through the food chain and water cycle, ultimately impacting human health (19). The generation of these air pollutants often shares common sources and processes with other pollutants (50). Low-carbon city pilots, while reducing carbon emissions, also achieve synergistic reductions in atmospheric pollutants, reducing the generation of wastewater and waste, thereby further improving public health. Based on the above analysis, this paper proposes the following hypotheses:

*H1*: Green low-carbon development have a significant positive effect on public health.

*H2*: Green low-carbon development can improve public health by mitigating climate change.

*H3*: Green low-carbon development can improve public health by reducing environmental pollution.

## 4 Study design

### 4.1 Model setting

To thoroughly investigate the impact of green low-carbon development on public health, this study used data from prefecture-level cities in China from 2007 to 2020. Leveraging the quasi-natural experiment provided by the low-carbon pilot city policy, we employed a DID model for empirical analysis. Cities were categorized into an experimental group (low-carbon pilot cities) and a control group (non-low-carbon pilot cities) based on their participation in the pilot program. The model specification is shown in Equation 1. When examining the mediation mechanism, the latest recommendations in causal inference suggest avoiding stepwise regression to prevent endogeneity issues (51). Therefore, we focused on the impact of the independent variable (low-carbon pilot cities) on the mediating variable (environmental pollution), assuming that the mediating variable directly affects the dependent variable (public health). The specific model is outlined in Equation (2).


(1) $$\mathrm{Phealt}h_{i,t}=a_{0}+a_{1}did_{i,t}+a_{2}\mathrm{Control}s_{i,t}+Year_{t}+City_{i}+e_{i,t}$$

(2) $$\mathrm{Pollutio}n_{i,t}=\beta_{0}+\beta_{1}did_{i,t}+\beta_{2}\mathrm{Control}s_{i,t}+year_{t}+City_{i}+\varepsilon_{i,t}$$

Here, 𝑖 represents the city and 𝑡 represents the year. *Phealth* and *Pollution* denote the public health level and environmental pollution in the city, respectively. The variable *did* is a dummy indicating pilot city status, 𝛼_0_ and 𝛽_0_ are constants, 𝛼_1_, 𝛼_2_, 𝛽_1_, 𝛽_2_ are coefficients to be estimated, *Controls* are control variables, *Year* and *City* represent fixed effects for years and cities, and e is the error term. The methods for measuring each variable in the model are described below.

### 4.2 Variable setting

#### 4.2.1 Dependent variable

Urban Public Health (*Phealth*). This study constructed a composite index of urban public health by combining essential support conditions with actual health performance improvements, based on the findings of Zeng Weiping (52). The foundation of public health, including the number of doctors per 10,000 people, *per capita* fiscal health expenditure, and the number of health institution beds per 10,000 people, represents the necessary inputs in terms of human, financial, and material resources. The goal is to maximize “expected outputs,” such as the survival rate, and minimize “undesired outputs,” such as the incidence of infectious diseases. The composite index of urban public health is calculated using the entropy method.

#### 4.2.2 Core independent variable

Green low-carbon development (*did*). Following Xie et al. (38), this dummy variable identifies whether a city is part of the low-carbon city pilot program. It equals 1 if the city was a pilot city for that year and subsequent years, and 0 otherwise.

#### 4.2.3 Mediating variables

Climate change (*CO₂*). This study measured climate change by a city’s carbon emission intensity, calculated as the total carbon emissions divided by the city’s GDP (40). Environmental pollution (*Pollution*) was measured by the industrial “three wastes” (wastewater, waste gas, and solid waste) (53). Due to missing solid waste data, we used industrial SO₂ emissions, industrial dust emissions, and PM2.5 as proxies for waste gas, and industrial wastewater emissions for wastewater.

#### 4.2.4 Control variables

Referring to the research of Chen (9) and Zhang (13, 14), this study controls for variables that may affect green and low-carbon development and public health, including city size, economic development, industrial structure, environment, education level, and openness. City size (*Size*) is the logged annual average population; economic development (*GDP*) is the logged *per capita* GDP; industrial structure (*Indus*) is the proportion of the secondary industry in GDP; environment (*Envir*) is the green coverage rate of the built-up area; education level (*Educa*) is the logged number of college students per 10,000 people; and openness (*Open*) is measured by travel activity (the ratio of total passenger traffic to the urban population).

### 4.3 Data sources and descriptive statistics

City information was taken from various editions of the “China Urban Statistical Yearbook,” and data on the incidence of infectious diseases were taken from the “China Health Statistics Yearbook.” Green finance data were sourced from the China CNRDS database. Missing values for public health were filled using linear interpolation, and samples with missing data for other variables were excluded. This resulted in 271 sample cities and 3,466 observations. The descriptive statistics are shown in Table 1.

**Table 1:** Descriptive statistics of variables.

Variable	*N*	Mean	SD	Min	p50	Max
*Phealth*	3,466	16.360	9.502	0.443	15.730	90.440
*did*	3,466	0.244	0.430	0.000	0.000	1.000
*Size*	3,466	5.886	0.715	−1.514	5.935	8.138
*GDP*	3,466	10.510	0.696	4.595	10.520	13.060
*Indus*	3,466	47.300	11.350	10.680	47.770	90.970
*Envir*	3,466	39.520	13.660	0.360	40.320	386.600
*Educa*	3,466	4.698	1.153	−0.211	4.670	8.570
*Open*	3,466	42.830	174.900	0.064	18.220	8234.000

## 5 Analysis of empirical results

### 5.1 Benchmark regression

The regression results for the impact of green low-carbon development on public health are presented in Table 2. Column (1) controls only for time and city fixed effects, while column (2) additionally includes control variables. In both regressions, the coefficients for “*did*” were 1.467 and 1.305, respectively, both significant at the 1% level. This indicates a substantial positive effect of green low-carbon development on public health, confirming the reasonableness of hypothesis 1.

**Table 2:** Benchmark regression results.

	(1)	(2)
Variables	Phealth	Phealth
*did*	1.467***	1.305***
	(5.912)	(5.902)
*Size*		−4.788***
		(−7.229)
*GDP*		−1.142***
		(−3.182)
*Indus*		−0.066***
		(−4.183)
*Envir*		0.001
		(0.275)
*Educa*		−0.048
		(−0.449)
*Open*		0.007***
		(13.864)
Observations	3,466	3,466
*R* ^2^	0.873	0.901

t-statistics in parentheses.*** *p* < 0.01, ** *p* < 0.05, * *p* < 0.1.

### 5.2 Robustness tests

#### 5.2.1 Parallel trend test

**Figure 4:**
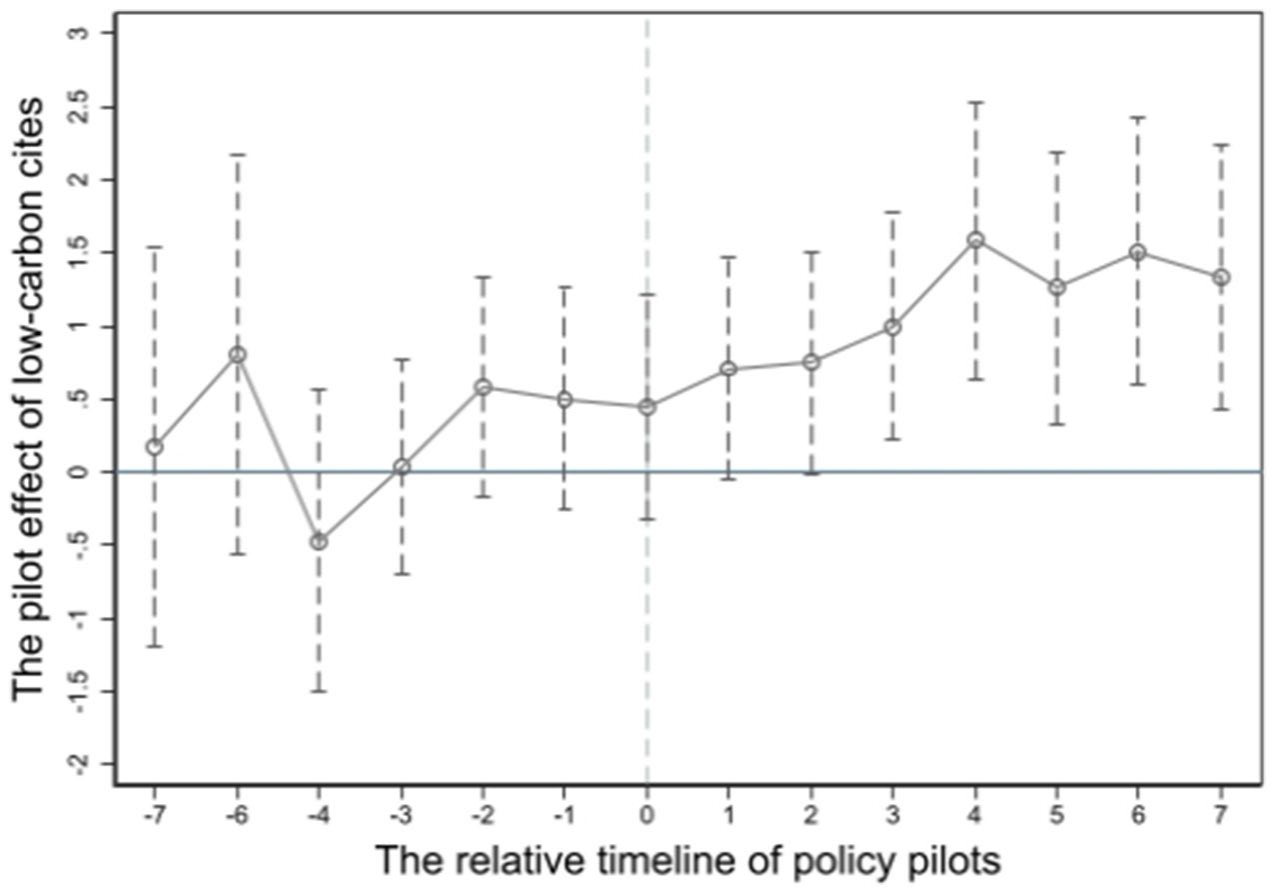
Parallel trend test plot.

A key requirement for using the DID model is the parallel trend assumption, which checks if the trends for the treatment and control groups were similar before the policy intervention. If the trends differ, it could bias the estimated policy effects. Following Zhang et al. (37), this study used an event study approach to test the parallel trend hypothesis. To this end, 14 dummy variables were defined based on the relative timing of policy implementation: *pre7* to *pre1* represent the 7 years to 1 year before policy implementation; *current* represents the implementation year; and *post1* to *post7* represent the 1–7 years after implementation. The *did* variable in model (1) is replaced with these dummy variables, using the fifth year before implementation as the reference period. The coefficients and 95% confidence intervals for each period are plotted in Figure 4. The *pre1* to *pre7* coefficients are all insignificant, indicating that pilot and non-pilot cities had similar trends before the policy, satisfying the parallel trend assumption. Additionally, the *post1* and *post2* coefficients are insignificant, while *post3* to *post7* are significantly positive, suggesting that green low-carbon development improve public health with a lag.

#### 5.2.2 Placebo test

**Figure 5:**
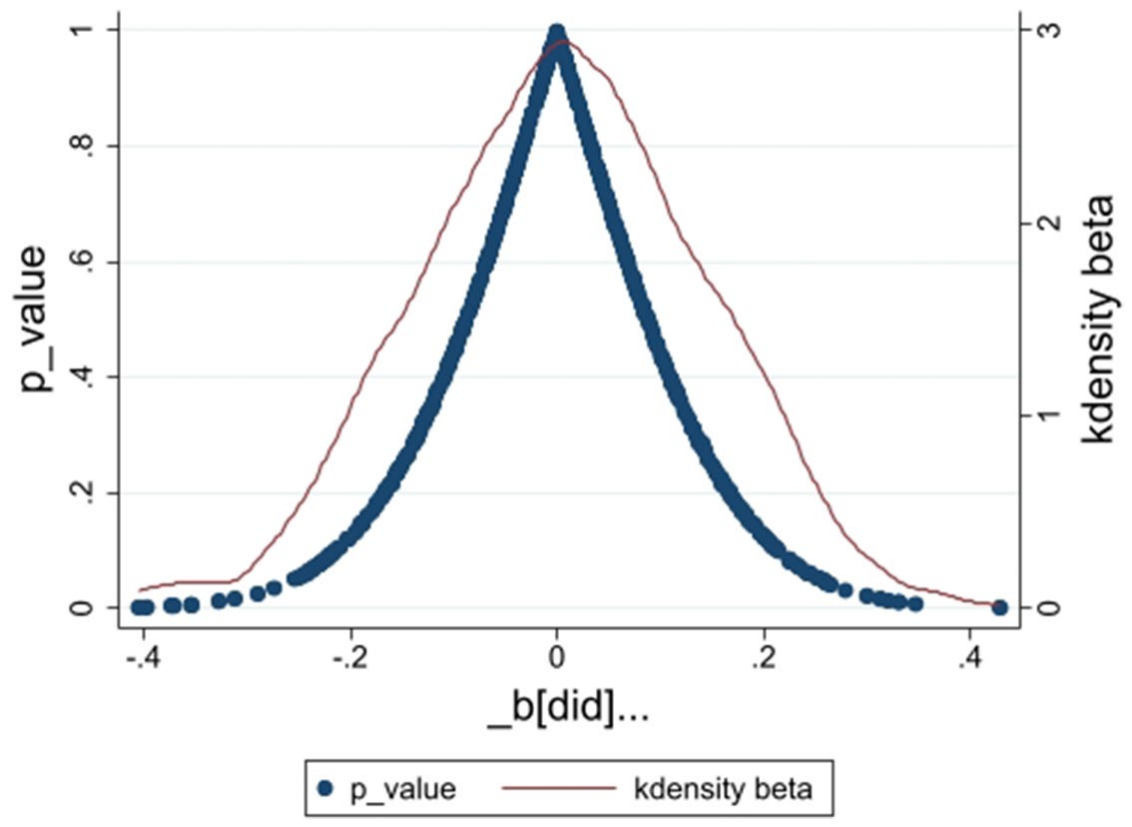
Placebo test plot.

To address potential biases from unobserved time-varying regional factors, we follow Shi et al. (44) for indirect verification. We randomly generated 500 “pseudo-policy” dummy variables based on the distribution of the *did* variable in the benchmark regression and re-estimated model (2). Figure 5 shows the distribution of 500 random coefficients and *p*-values. The “pseudo-policy” coefficients approximate a normal distribution centered around zero, with most *p*-values exceeding 0.10, indicating no significance. This suggests that the positive effects of green low-carbon development on public health are not due to random factors.

#### 5.2.3 Sample selection bias

To address potential sample selection bias due to the limited number of low-carbon pilot cities, we used the propensity score matching difference-in-differences (PSM-DID) method for robustness testing. Following Qiao et al. (54), we constructed a logit model using control variables from model (1) for 1:3 nearest neighbor matching with replacement, pairing each pilot city with the most similar non-pilot city. This ensures that cities in both groups are comparable except for the policy impact. After excluding unmatched samples, regression analysis was conducted on the matched dataset. Column (1) of Table 3 shows that the *did* coefficient was significantly positive at the 1% level, indicating no serious sample selection bias and confirming the robustness of the results.

**Table 3:** Robustness test results.

	(1)	(2)	(3)	(4)
Variables	Phealth	Phealth	Phealth	Phealth
*did*	0.588**	1.274***	1.108***	1.025***
	(2.207)	(5.775)	(3.797)	(4.941)
*Size*	−3.774***	−5.251***	−5.330***	−3.229***
	(−3.097)	(−7.848)	(−4.800)	(−4.842)
*GDP*	−1.048***	−1.097***	−0.862*	−0.932***
	(−2.706)	(−3.064)	(−1.772)	(−2.772)
*Indus*	−0.063***	−0.066***	−0.116***	−0.051***
	(−3.167)	(−4.230)	(−5.560)	(−3.456)
*Envir*	0.030**	0.002	0.002	0.001
	(2.401)	(0.415)	(0.356)	(0.257)
*Educa*	−0.132	−0.035	−0.183	0.012
	(−0.910)	(−0.326)	(−1.373)	(0.125)
*Open*	0.004***	0.007***	0.007***	0.008***
	(3.983)	(12.644)	(8.107)	(15.092)
Density		0.005***		
		(4.344)		
Observations	3,007	3,463	2,560	3,407
*R*-squared	0.870	0.901	0.905	0.894

Standard errors in parentheses. *** *p* < 0.01, ** *p* < 0.05, * *p* < 0.1.

#### 5.2.4 Other robustness tests

To conduct other robustness tests, we first added control variables. Population density is another key factor affecting public health (55). Therefore, we included population density (*Density*) as an additional control variable. Column (2) of Table 3 shows that the *did* coefficient remained significantly positive at the 1% level. Second, we changed the sample period. By focusing on the first two batches of low-carbon pilot cities from 2007 to 2016, we tested their impact on public health. Column (3) of Table 3 shows that the *did* coefficient was significantly positive at the 1% level. Third, we changed the sample spatial range. Excluding municipalities, which have distinct policy environments and resources, column (4) of Table 3 shows that the *did* coefficient was significantly positive at the 1% level. These robustness tests collectively support the study’s hypotheses.

### 5.3 Mechanism testing

Based on the theoretical analysis, we empirically tested the mediating effects of green low-carbon development on public health. Table 4 reports the mediation test results. Column (1) shows that the *did* coefficient was significantly negative at the 1% level, indicating that green low-carbon development reduce carbon emission intensity, which is consistent with the empirical outcomes of previous research (8). Column (2) shows that the *did* coefficient was significantly negative at the 5% level, indicating a reduction in PM2.5 levels, This finding further supports the inhibitory impact of green low-carbon development on air pollution, particularly PM2.5 (9). Column (3) shows that the *did* coefficient was significantly negative at the 1% level, indicating a reduction in sulfur dioxide emissions. Column (4) shows that the *did* coefficient was significantly positive at the 5% level, indicating a reduction in industrial dust emissions. Column (5) shows that the *did* coefficient was significantly positive at the 5% level, indicating a reduction in industrial wastewater emissions. The results of columns (3)–(5) clearly indicate that this study offers a more comprehensive analysis of environmental pollution. It not only explores the impact of green low-carbon development on haze pollution but also considers its effects on sulfur dioxide, wastewater, and smoke emissions, refining previous research findings (19, 26).

**Table 4:** Mediation test results.

	(1)	(2)	(3)	(4)	(5)
Variables	CO_2_	PM2.5	SO_2_	Dust	Wastewater
*did*	−0.033***	−0.028***	−0.179***	−0.108**	−0.120***
	(0.008)	(0.005)	(0.038)	(0.050)	(0.026)
*Size*	−0.421***	−0.068***	−0.125	−0.016	−0.044
	(0.025)	(0.016)	(0.114)	(0.149)	(0.078)
*GDP*	−0.525***	−0.067***	0.141**	0.017	0.038
	(0.014)	(0.008)	(0.062)	(0.081)	(0.042)
*Indus*	−0.008***	−0.000	0.001	0.000	−0.002
	(0.001)	(0.000)	(0.003)	(0.004)	(0.002)
*Envir*	−0.001***	0.000	0.002*	−0.001	0.001
	(0.000)	(0.000)	(0.001)	(0.001)	(0.001)
*Educa*	−0.009**	0.004*	0.048***	−0.017	−0.009
	(0.004)	(0.003)	(0.019)	(0.024)	(0.013)
*Open*	−0.000***	−0.000***	0.000	−0.000	−0.000
	(0.000)	(0.000)	(0.000)	(0.000)	(0.000)
Observations	3,463	3,463	3,449	3,424	3,463
*R*-squared	0.886	0.962	0.871	0.816	0.828

Standard errors in parentheses. *** *p* < 0.01, ** *p* < 0.05, * *p* < 0.1.

Table 5 shows the impacts of climate change and environmental pollution on public health, with coefficients in columns (1) to (5) all significantly negative, further confirming the adverse effects of climate change and environmental pollution on public health. These findings are in line with the research results of Ebi et al. (45) and Clayton (23), suggesting that environmental pollution exacerbates living conditions and the occurrence of extreme weather increases the likelihood of mortality, posing a threat to public safety and health. However, the results of Tables 4, 5 indicate that low-carbon city construction can improve public health by mitigating climate change and reducing environmental pollution, and hypotheses 2 and 3 have been validated. Low-carbon green development can effectively reduce carbon emissions, enhance ecological civilization efforts, mitigate climate change, improve national health literacy, and increase overall happiness.

**Table 5:** Impact of climate change and environmental pollution on public health.

	(4)	(5)	(6)	(7)	(8)
Variables	Phealth	Phealth	Phealth	Phealth	Phealth
*CO_2_*	−4.728***				
	(0.458)				
*PM2.5*		−1.832**			
		(0.749)			
*SO_2_*			−0.020***		
			(0.004)		
*Dust*				−0.156**	
				(0.077)	
*Wastewater*					−1.717***
					(0.148)
*Size*	−6.739***	−4.872***	−4.918***	−4.942***	−4.828***
	(0.683)	(0.667)	(0.646)	(0.647)	(0.652)
*GDP*	−3.593***	−1.231***	−0.839**	−0.967***	−1.047***
	(0.429)	(0.364)	(0.351)	(0.350)	(0.353)
*Indus*	−0.102***	−0.064***	−0.056***	−0.060***	−0.067***
	(0.016)	(0.016)	(0.015)	(0.015)	(0.015)
*Envir*	−0.003	0.000	0.001	−0.000	0.001
	(0.005)	(0.005)	(0.005)	(0.005)	(0.005)
*Educa*	−0.059	−0.006	0.003	−0.002	−0.033
	(0.106)	(0.107)	(0.104)	(0.104)	(0.105)
*Open*	0.006***	0.007***	0.007***	0.007***	0.007***
	(0.001)	(0.001)	(0.001)	(0.001)	(0.001)
Observations	3,463	3,463	3,449	3,424	3,463
*R*-squared	0.903	0.900	0.899	0.898	0.903

Standard errors in parentheses. *** *p* < 0.01, ** *p* < 0.05, * *p* < 0.1.

### 5.4 Heterogeneity analysis

#### 5.4.1 Heterogeneity analysis by city tier

City tiers reflect various factors such as administrative level, economic scale, and social development. Different tiers may have significant differences in resources, policy priorities, and support. This study categorized the sample cities into first-and second-tier cities and other cities based on the classification method of Ni (56) and then performed separate regressions. The results are shown in columns (1) and (2) of Table 6. The regression coefficient for first-and second-tier cities was 1.469, significant at the 1% level, while the coefficient for other cities was not significant. These results indicate that the positive impact of low-carbon pilot cities on public health is more pronounced in first-and second-tier cities. This could be due to the more advanced infrastructure and resources available in these cities, which facilitate the effective implementation and maintenance of low-carbon projects. Additionally, the higher environmental awareness and participation in developed cities likely support and enhance low-carbon lifestyle adoption, improving the pilot projects’ effectiveness.

**Table 6:** Heterogeneity analysis.

	City tier		Economic growth		Technological investment		Green finance	
Variables	(1)	(2)	(3)	(4)	(5)	(6)	(7)	(8)
	Phealth	Phealth	Phealth	Phealth	Phealth	Phealth	Phealth	Phealth
*did*	1.469***	0.178	0.782**	0.121	2.349***	−0.322	1.543***	0.219
	(0.541)	(0.199)	(0.375)	(0.271)	(0.294)	(0.313)	(0.317)	(0.367)
*Size*	−14.418***	−4.215***	−5.981***	−4.819***	−7.858***	−4.676***	−9.600***	−1.927*
	(1.546)	(0.631)	(1.555)	(1.093)	(1.005)	(1.297)	(1.244)	(1.060)
*GDP*	3.231***	−0.966***	−0.228	−1.755***	−0.666	−1.063**	−0.852	−0.477
	(1.054)	(0.317)	(0.623)	(0.417)	(0.550)	(0.440)	(0.564)	(0.473)
*Indus*	−0.364***	−0.022	−0.159***	0.001	−0.036	−0.046**	−0.051**	−0.069***
	(0.067)	(0.013)	(0.032)	(0.017)	(0.027)	(0.019)	(0.024)	(0.023)
*Envir*	−0.001	0.013	0.039***	−0.004	0.003	0.000	0.008	0.019
	(0.005)	(0.009)	(0.010)	(0.005)	(0.005)	(0.011)	(0.006)	(0.014)
*Educa*	0.294	0.042	−0.450**	−0.059	0.370**	0.001	−0.122	0.195
	(0.419)	(0.092)	(0.198)	(0.126)	(0.175)	(0.126)	(0.157)	(0.139)
*Open*	0.006***	0.007***	0.006***	−0.001	0.004***	0.007***	0.005***	0.009***
	(0.001)	(0.000)	(0.001)	(0.001)	(0.001)	(0.001)	(0.001)	(0.001)
Observations	316	3,091	1,725	1,695	1,720	1,720	1,721	1,706
*R*-squared	0.956	0.880	0.896	0.951	0.935	0.883	0.920	0.896

Standard errors in parentheses. *** *p* < 0.01, ** *p* < 0.05, * *p* < 0.1.

#### 5.4.2 Heterogeneity analysis by economic growth

The level of economic growth in a city can indicate its vitality and attractiveness. This study divided the sample cities into high and low economic growth groups based on the median regional GDP growth rate and conducted separate regressions. The results are shown in columns (3) and (4) of Table 6. The regression coefficient for high-growth cities was 0.78, significant at the 5% level, while the coefficient for low-growth cities was not significant. These findings suggest that low-carbon pilot cities have a more significant positive impact on public health in high-growth cities. This may be because these cities can attract more talent and advanced technologies, which are crucial for developing and applying low-carbon innovations.

#### 5.4.3 Heterogeneity analysis by technological investment

Technological investment is crucial for advancing low-carbon technologies and solutions. Cities with different levels of investment in research and application will experience varying effects on the development of low-carbon technologies, in turn impacting public health improvements. This study categorized the sample cities into high and low technological investment groups based on the median investment amount and performed separate regressions. The results are shown in columns (5) and (6) of Table 6. The regression coefficient for high technological investment cities was 2.349, significant at the 1% level, while the coefficient for low technological investment cities was not significant. These findings indicate that low-carbon pilot cities have a more significant positive impact on public health in high technological investment cities. This may be because higher technological investment drives innovation in low-carbon technologies, allowing these cities to more effectively reduce pollution, improve air quality, and promote public health.

#### 5.4.4 Heterogeneity analysis by green finance

Green finance supports environmentally friendly projects and technologies through financial products and services. The development level of green finance in a city affects the funding and sustainability of low-carbon projects. Following the method of Liu and He Chun (57), this study constructed a green finance index and categorized the sample cities into high and low green finance groups based on the median index value, then conducted separate regressions. The results are shown in columns (7) and (8) of Table 6. The regression coefficient for high green finance cities was 1.543, significant at the 1% level, while the coefficient for low green finance cities was not significant. These findings indicate that low-carbon pilot cities have a more significant positive impact on public health in high green finance cities. This may be because a robust green finance system provides the necessary funding for low-carbon projects, facilitating the implementation of sustainable development initiatives.

## 6 Conclusion and policy recommendations

### 6.1 Conclusion

Green low-carbon development is essential for achieving carbon peaking, carbon neutrality, and the “Healthy China” strategy. Selecting suitable environmental policy tools and establishing a comprehensive “dual carbon” policy framework are critical for prioritizing public health and fostering harmonious environmental and human development. Using the low-carbon city pilot policy as a quasi-natural experiment, this study employed a multi-period DID model to analyze the impact of urban green low-carbon development on public health and its underlying mechanisms. The results indicate that urban green low-carbon development positively impacts public health. After endogeneity concerns are addressed and robustness checks are conducted, this effect remains consistent. This extends the findings of Cheng et al. (10), Song et al. (31), and Zhang & Zheng (37), showing that low-carbon city initiatives not only improve ecological quality and encourage green lifestyles, but also boost public health.

Mechanism analysis reveals that low-carbon city pilots improve public health by mitigating climate change and reducing environmental pollution. This supports the findings of Babuji et al. (13), Clayton (23), and Hallegatte et al. (47), who assert that climate change and environmental pollution adversely affect public health. This study innovatively identifies climate change and environmental pollution as mediating pathways through which green low-carbon development enhances public health. Low-carbon city pilots reduce urban carbon emission intensity and decrease emissions of PM2.5, SO₂, industrial dust, and wastewater, thus mitigating climate change and reducing environmental pollution, which in turn improves public health.

Heterogeneity analysis shows that the impact of low-carbon city pilots on public health varies by city tier, economic growth, technological investment, and green finance. The effects are more pronounced in first-and second-tier cities, as well as in cities with rapid economic growth, high technological investment, and well-developed green finance systems. These differences may be due to stronger environmental policy enforcement, ample research funding, diverse and accessible green finance channels, and effective clean production and industrial upgrading in these cities. In contrast, cities with weaker policy enforcement and insufficient technological and industrial capabilities see less impact from low-carbon initiatives.

The conclusions of this paper support previous research highlighting the effectiveness of low-carbon pilot cities in improving public health and addressing the research gap on how green low-carbon development can enhance public health. These findings not only provide empirical support for developing low-carbon pilot cities but also offer valuable insights for future policy-making, particularly in advancing sustainable development and environmental protection.

Although this study has achieved certain research outcomes, it also has some limitations. To address these, future research will focus on the following areas. First of all, expand the geographical scope of the research sample to include a global perspective. Achieving carbon net-zero emissions and promoting regional sustainable development are worldwide challenges. This study focuses on the effects of low-carbon city pilot policies in developing countries, with China as a representative case. However, developed countries vary in economic development levels and environmental regulations. Therefore, future research will compile a global dataset of green low-carbon development policies and conduct comparative analyses to generate more diverse and comprehensive research findings. In addition, this study only explores the effects of green low-carbon city pilots on public health. Currently, China has implemented pilot zones for green finance reform, climate-adaptive city construction, and new energy policies. Future research could explore the economic effects of green transition and the policy synergy resulting from combining low-carbon city construction with other policies.

### 6.2 Policy recommendations

First, it is important to prioritize high-quality development in low-carbon city initiatives to maximize their public health benefits. Governments should expand the scope of low-carbon city pilots, invest in green infrastructure, increase technological investment, and enhance green financial services to stimulate urban green development. A comprehensive approach should be adopted to assess the environmental governance effects of low-carbon cities and to understand the impact of carbon reduction and pollution control on public health. Best practices from existing low-carbon city pilots should be summarized and promoted to create a green and healthy environment for the public.

Second, it is important to aim for environmental health equity in low-carbon city construction by establishing a multi-stakeholder collaborative governance system. Environmental policies should be tailored to the specific needs of different regions and city sizes. Policies should synergize with supplementary measures such as medical insurance benefits and ecological compensation. A collaborative governance system involving multiple departments and regions should be developed to facilitate green technology sharing among cities of different sizes and development levels. This will help achieve environmental health equity and create a harmonious and inclusive “Healthy China.”

Third, it is critical that businesses and individuals are encouraged to actively participate in low-carbon city initiatives and to raise their environmental awareness. Businesses should commit to the dual goals of carbon peaking and carbon neutrality, forming green technology research teams, innovating production processes, reducing waste and carbon emissions, and minimizing environmental impacts. This will contribute to efficient green productivity and sustainable development. Individuals should increase their awareness of environmental issues, recognize the health risks of environmental degradation, and adopt green consumption and travel habits.

## Data Availability

The original contributions presented in the study are included in the article/Supplementary material, further inquiries can be directed to the corresponding author/s.

## Footnotes

^1^Data source: https://www.mee.gov.cn/hjzl/sthjzk/zghjzkgb/202406/P020240604551536165161.pdf

^2^“Notice on Launching Low-Carbon Province and Low-Carbon City Pilot Projects” by the National Development and Reform Commission. https://www.ndrc.gov.cn/xxgk/zcfb/tz/201008/t20100810_964674.html. First batch of pilots: Guangdong Province, Liaoning Province, Hubei Province, Shaanxi Province, Yunnan Province, Tianjin, Chongqing, Shenzhen, Xiamen, Hangzhou, Nanchang, Guiyang, Baoding.

^3^Second batch of pilot cities: Shijiazhuang, Qinhuangdao, Jincheng, Hulunbuir, Jilin, Daxing’anling, Suzhou, Huai’an, Zhenjiang, Ningbo, Wenzhou, Chizhou, Nanping, Jingdezhen, Ganzhou, Qingdao, Jiyuan, Wuhan, Guangzhou, Guilin, Guangyuan, Zunyi, Kunming, Yan’an, Jinchang, Urumqi. Third batch of pilot cities: Nanjing, Changzhou, Wuhai, Shenyang, Dalian, Chaoyang, Xunke County, Jiaxing, Jinhua, Quzhou, Hefei, Huaibei, Huangshan, Lu′an, Xuancheng, Sanming, Gongqingcheng, Ji’an, Fuzhou, Jinan, Yantai, Weifang, Changyang Tujia Autonomous County, Changsha, Zhuzhou, Xiangtan, Chenzhou, Zhongshan, Liuzhou, Sanya, Qiongzhong Li and Miao Autonomous County, Chengdu, Yuxi, Simao District of Pu′er, Lhasa, Ankang, Lanzhou, Dunhuang, Xining, Yinchuan, Wuzhong, Changji, Yining, Hotan, Alar City of the First Division.
