# Supplementary material for: The impact of green low-carbon development on public health: a quasi-natural experimental study of low-carbon pilot cities in China
Source: Front Public Health. 2024 Oct 8;12:1470592. doi: 10.3389/fpubh.2024.1470592 (PMC11493735; doi:10.3389/fpubh.2024.1470592)
Supplement: Supplementary file 1 [file Data_Sheet_1.docx]

Supplementary Material

# Supplementary Figures

**
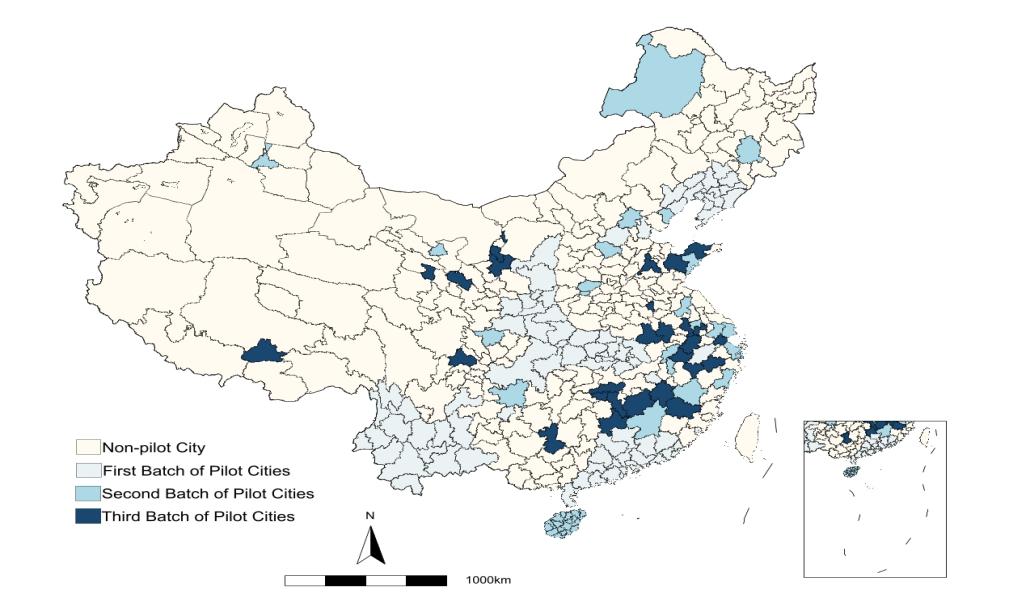
**

**Supplementary Figure 1.** Distribution map of low-carbon pilot cities in China


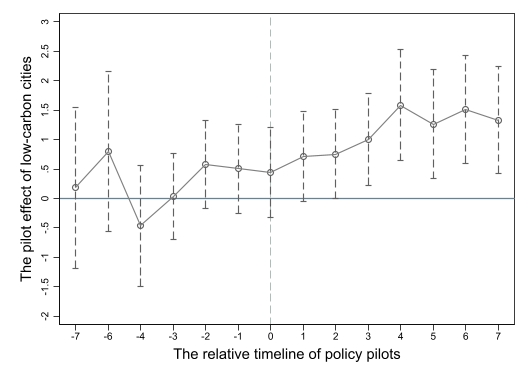


**Supplementary Figure 2.** Parallel trend test plot


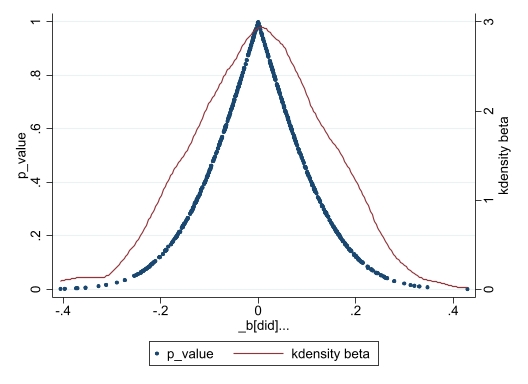


**Supplementary Figure 3.** Placebo test plot

# Supplementary Tables

**Supplementary Table 1.** Descriptive statistics of variables

| Variable | N | Mean | SD | Min | p50 | Max |
| --- | --- | --- | --- | --- | --- | --- |
| Phealth | 3466.000 | 16.360 | 9.502 | 0.443 | 15.730 | 90.440 |
| did | 3466.000 | 0.244 | 0.430 | 0.000 | 0.000 | 1.000 |
| Size | 3466.000 | 5.886 | 0.715 | -1.514 | 5.935 | 8.138 |
| GDP | 3466.000 | 10.510 | 0.696 | 4.595 | 10.520 | 13.060 |
| Indus | 3466.000 | 47.300 | 11.350 | 10.680 | 47.770 | 90.970 |
| Envir | 3466.000 | 39.520 | 13.660 | 0.360 | 40.320 | 386.600 |
| Educa | 3463.000 | 4.698 | 1.153 | -0.211 | 4.670 | 8.570 |
| Open | 3466.000 | 42.830 | 174.900 | 0.064 | 18.220 | 8234.000 |

**Supplementary Table 2.** Benchmark regression results

|  | (1) | (2) |
| --- | --- | --- |
| VARIABLES | Phealth | Phealth |
| did | 1.467*** | 1.305*** |
|  | (5.912) | (5.902) |
| Size |  | -4.788*** |
|  |  | (-7.229) |
| GDP |  | -1.142*** |
|  |  | (-3.182) |
| Indus |  | -0.066*** |
|  |  | (-4.183) |
| Envir |  | 0.001 |
|  |  | (0.275) |
| Educa |  | -0.048 |
|  |  | (-0.449) |
| Open |  | 0.007*** |
|  |  | (13.864) |
| Observations | 3,466 | 3,463 |
| R-squared | 0.873 | 0.901 |

Note：t-statistics in parentheses

*** p<0.01, ** p<0.05, * p<0.1

**Supplementary Table 3.** Robustness test results

|  | (1) | (2) | (3) | (4) |
| --- | --- | --- | --- | --- |
| VARIABLES | Phealth | Phealth | Phealth | Phealth |
| did | 0.588** | 1.274*** | 1.108*** | 1.025*** |
|  | (2.207) | (5.775) | (3.797) | (4.941) |
| Size | -3.774*** | -5.251*** | -5.330*** | -3.229*** |
|  | (-3.097) | (-7.848) | (-4.800) | (-4.842) |
| GDP | -1.048*** | -1.097*** | -0.862* | -0.932*** |
|  | (-2.706) | (-3.064) | (-1.772) | (-2.772) |
| Indus | -0.063*** | -0.066*** | -0.116*** | -0.051*** |
|  | (-3.167) | (-4.230) | (-5.560) | (-3.456) |
| Envir | 0.030** | 0.002 | 0.002 | 0.001 |
|  | (2.401) | (0.415) | (0.356) | (0.257) |
| Educa | -0.132 | -0.035 | -0.183 | 0.012 |
|  | (-0.910) | (-0.326) | (-1.373) | (0.125) |
| Open | 0.004*** | 0.007*** | 0.007*** | 0.008*** |
|  | (3.983) | (12.644) | (8.107) | (15.092) |
| Density |  | 0.005*** |  |  |
|  |  | (4.344) |  |  |
| Observations | 3,007 | 3,463 | 2,560 | 3,407 |
| R-squared | 0.870 | 0.901 | 0.905 | 0.894 |

**Supplementary Table 4.** Mediation test results

|  | (1) | (2) | (3) | (4) | （5） |
| --- | --- | --- | --- | --- | --- |
| VARIABLES | CO_2_ | PM2.5 | SO_2_ | Dust | Wastewater |
| did | -0.033*** | -0.028*** | -0.179*** | -0.108** | -0.120*** |
|  | (0.008) | (0.005) | (0.038) | (0.050) | (0.026) |
| Size | -0.421*** | -0.068*** | -0.125 | -0.016 | -0.044 |
|  | (0.025) | (0.016) | (0.114) | (0.149) | (0.078) |
| GDP | -0.525*** | -0.067*** | 0.141** | 0.017 | 0.038 |
|  | (0.014) | (0.008) | (0.062) | (0.081) | (0.042) |
| Indus | -0.008*** | -0.000 | 0.001 | 0.000 | -0.002 |
|  | (0.001) | (0.000) | (0.003) | (0.004) | (0.002) |
| Envir | -0.001*** | 0.000 | 0.002* | -0.001 | 0.001 |
|  | (0.000) | (0.000) | (0.001) | (0.001) | (0.001) |
| Educa | -0.009** | 0.004* | 0.048*** | -0.017 | -0.009 |
|  | (0.004) | (0.003) | (0.019) | (0.024) | (0.013) |
| Open | -0.000*** | -0.000*** | 0.000 | -0.000 | -0.000 |
|  | (0.000) | (0.000) | (0.000) | (0.000) | (0.000) |
| Observations | 3,463 | 3,463 | 3,449 | 3,424 | 3,463 |
| R-squared | 0.886 | 0.962 | 0.871 | 0.816 | 0.828 |

**Supplementary Table 5.** Impact of climate change and environmental pollution on public health

|  | (4) | (5) | (6) | (7) | (8) |
| --- | --- | --- | --- | --- | --- |
| VARIABLES | Phealth | Phealth | Phealth | Phealth | Phealth |
| CO_2_ | -4.728*** |  |  |  |  |
|  | (0.458) |  |  |  |  |
| PM2.5 |  | -1.832** |  |  |  |
|  |  | (0.749) |  |  |  |
| SO_2_ |  |  | -0.020*** |  |  |
|  |  |  | (0.004) |  |  |
| Dust |  |  |  | -0.156** |  |
|  |  |  |  | (0.077) |  |
| Wastewater |  |  |  |  | -1.717*** |
|  |  |  |  |  | (0.148) |
| Size | -6.739*** | -4.872*** | -4.918*** | -4.942*** | -4.828*** |
|  | (0.683) | (0.667) | (0.646) | (0.647) | (0.652) |
| GDP | -3.593*** | -1.231*** | -0.839** | -0.967*** | -1.047*** |
|  | (0.429) | (0.364) | (0.351) | (0.350) | (0.353) |
| Indus | -0.102*** | -0.064*** | -0.056*** | -0.060*** | -0.067*** |
|  | (0.016) | (0.016) | (0.015) | (0.015) | (0.015) |
| Envir | -0.003 | 0.000 | 0.001 | -0.000 | 0.001 |
|  | (0.005) | (0.005) | (0.005) | (0.005) | (0.005) |
| Educa | -0.059 | -0.006 | 0.003 | -0.002 | -0.033 |
|  | (0.106) | (0.107) | (0.104) | (0.104) | (0.105) |
| Open | 0.006*** | 0.007*** | 0.007*** | 0.007*** | 0.007*** |
|  | (0.001) | (0.001) | (0.001) | (0.001) | (0.001) |
| Observations | 3,463 | 3,463 | 3,449 | 3,424 | 3,463 |
| R-squared | 0.903 | 0.900 | 0.899 | 0.898 | 0.903 |

**Supplementary Table 6.** Heterogeneity analysis

|  | City tier | | Economic growth | | Technological investment | | Green finance | |
| --- | --- | --- | --- | --- | --- | --- | --- | --- |
| VARIABLES | (1) | (2) | (3) | (4) | (5) | (6) | (7) | (8) |
|  | Phealth | Phealth | Phealth | Phealth | Phealth | Phealth | Phealth | Phealth |
| did | 1.469*** | 0.178 | 0.782** | 0.121 | 2.349*** | -0.322 | 1.543*** | 0.219 |
|  | (0.541) | (0.199) | (0.375) | (0.271) | (0.294) | (0.313) | (0.317) | (0.367) |
| Size | -14.418*** | -4.215*** | -5.981*** | -4.819*** | -7.858*** | -4.676*** | -9.600*** | -1.927* |
|  | (1.546) | (0.631) | (1.555) | (1.093) | (1.005) | (1.297) | (1.244) | (1.060) |
| GDP | 3.231*** | -0.966*** | -0.228 | -1.755*** | -0.666 | -1.063** | -0.852 | -0.477 |
|  | (1.054) | (0.317) | (0.623) | (0.417) | (0.550) | (0.440) | (0.564) | (0.473) |
| Indus | -0.364*** | -0.022 | -0.159*** | 0.001 | -0.036 | -0.046** | -0.051** | -0.069*** |
|  | (0.067) | (0.013) | (0.032) | (0.017) | (0.027) | (0.019) | (0.024) | (0.023) |
| Envir | -0.001 | 0.013 | 0.039*** | -0.004 | 0.003 | 0.000 | 0.008 | 0.019 |
|  | (0.005) | (0.009) | (0.010) | (0.005) | (0.005) | (0.011) | (0.006) | (0.014) |
| Educa | 0.294 | 0.042 | -0.450** | -0.059 | 0.370** | 0.001 | -0.122 | 0.195 |
|  | (0.419) | (0.092) | (0.198) | (0.126) | (0.175) | (0.126) | (0.157) | (0.139) |
| Open | 0.006*** | 0.007*** | 0.006*** | -0.001 | 0.004*** | 0.007*** | 0.005*** | 0.009*** |
|  | (0.001) | (0.000) | (0.001) | (0.001) | (0.001) | (0.001) | (0.001) | (0.001) |
| Observations | 316 | 3,091 | 1,725 | 1,695 | 1,720 | 1,720 | 1,721 | 1,706 |
| R-squared | 0.956 | 0.880 | 0.896 | 0.951 | 0.935 | 0.883 | 0.920 | 0.896 |
