# Supplementary material for: The impact of green low-carbon development on public health: a quasi-natural experimental study of low-carbon pilot cities in China
Source: Front Public Health. 2024 Oct 8;12:1470592. doi: 10.3389/fpubh.2024.1470592 (PMC11493735; doi:10.3389/fpubh.2024.1470592)
Supplement: Supplementary file 2 [file Data_Sheet_1.ZIP › Code,data and results/Figures and Tables/PSM-DID.doc]

	(1)	
VARIABLES	y	
		
did	0.588**	
	(2.207)	
Size	-3.774***	
	(-3.097)	
GDP	-1.048***	
	(-2.706)	
Indus	-0.063***	
	(-3.167)	
Envir	0.030**	
	(2.401)	
Educa	-0.132	
	(-0.910)	
Open	0.004***	
	(3.983)	
		
Observations	3,007	
R-squared	0.870	
t-statistics in parentheses
*** p<0.01, ** p<0.05, * p<0.1
