# Supplementary material for: The impact of green low-carbon development on public health: a quasi-natural experimental study of low-carbon pilot cities in China
Source: Front Public Health. 2024 Oct 8;12:1470592. doi: 10.3389/fpubh.2024.1470592 (PMC11493735; doi:10.3389/fpubh.2024.1470592)
Supplement: Supplementary file 2 [file Data_Sheet_1.ZIP › Code,data and results/Figures and Tables/results.doc]

Wastewater
	(1)	(2)	(3)	(4)	
VARIABLES	pm25	SO2	Dust	wastewater	
					
did	-0.0281***	-0.179***	-0.108**	-0.120***	
	(0.00524)	(0.0384)	(0.0501)	(0.0260)	
Size	-0.0682***	-0.125	-0.0159	-0.0438	
	(0.0157)	(0.114)	(0.149)	(0.0778)	
GDP	-0.0674***	0.141**	0.0174	0.0377	
	(0.00850)	(0.0621)	(0.0807)	(0.0422)	
Indus	-0.000324	0.000790	6.88e-05	-0.00237	
	(0.000373)	(0.00272)	(0.00354)	(0.00185)	
Envir	0.000119	0.00169*	-0.000535	0.000567	
	(0.000127)	(0.000927)	(0.00120)	(0.000630)	
Educa	0.00420*	0.0480***	-0.0175	-0.00898	
	(0.00254)	(0.0185)	(0.0241)	(0.0126)	
Open	-5.60e-05***	4.85e-06	-3.65e-05	-3.19e-05	
	(1.22e-05)	(8.91e-05)	(0.000116)	(6.05e-05)	
					
Observations	3,463	3,449	3,424	3,463	
R-squared	0.962	0.871	0.816	0.828	
Standard errors in parentheses
*** p<0.01, ** p<0.05, * p<0.1
