# Supplementary material for: The impact of green low-carbon development on public health: a quasi-natural experimental study of low-carbon pilot cities in China
Source: Front Public Health. 2024 Oct 8;12:1470592. doi: 10.3389/fpubh.2024.1470592 (PMC11493735; doi:10.3389/fpubh.2024.1470592)
Supplement: Supplementary file 2 [file Data_Sheet_1.ZIP › Code,data and results/Figures and Tables/中介.doc]

Wastewater Regression
	(1)	(2)	(3)	(4)	
VARIABLES	pm25	SO2	Dust	wastewater	
					
did	-0.028***	-0.179***	-0.108**	-0.120***	
	(0.005)	(0.038)	(0.050)	(0.026)	
Size	-0.068***	-0.125	-0.016	-0.044	
	(0.016)	(0.114)	(0.149)	(0.078)	
GDP	-0.067***	0.141**	0.017	0.038	
	(0.008)	(0.062)	(0.081)	(0.042)	
Indus	-0.000	0.001	0.000	-0.002	
	(0.000)	(0.003)	(0.004)	(0.002)	
Envir	0.000	0.002*	-0.001	0.001	
	(0.000)	(0.001)	(0.001)	(0.001)	
Educa	0.004*	0.048***	-0.017	-0.009	
	(0.003)	(0.019)	(0.024)	(0.013)	
Open	-0.000***	0.000	-0.000	-0.000	
	(0.000)	(0.000)	(0.000)	(0.000)	
					
Observations	3,463	3,449	3,424	3,463	
R-squared	0.962	0.871	0.816	0.828	
Standard errors in parentheses
*** p<0.01, ** p<0.05, * p<0.1
