# Supplementary material for: The impact of green low-carbon development on public health: a quasi-natural experimental study of low-carbon pilot cities in China
Source: Front Public Health. 2024 Oct 8;12:1470592. doi: 10.3389/fpubh.2024.1470592 (PMC11493735; doi:10.3389/fpubh.2024.1470592)
Supplement: Supplementary file 2 [file Data_Sheet_1.ZIP › Code,data and results/Figures and Tables/修改样本时间范围.doc]

	(1)	
VARIABLES	y	
		
did	1.108***	
	(3.797)	
Size	-5.330***	
	(-4.800)	
GDP	-0.862*	
	(-1.772)	
Indus	-0.116***	
	(-5.560)	
Envir	0.002	
	(0.356)	
Educa	-0.183	
	(-1.373)	
Open	0.007***	
	(8.107)	
		
Observations	2,560	
R-squared	0.905	
t-statistics in parentheses
*** p<0.01, ** p<0.05, * p<0.1
