# Supplementary material for: The impact of green low-carbon development on public health: a quasi-natural experimental study of low-carbon pilot cities in China
Source: Front Public Health. 2024 Oct 8;12:1470592. doi: 10.3389/fpubh.2024.1470592 (PMC11493735; doi:10.3389/fpubh.2024.1470592)
Supplement: Supplementary file 2 [file Data_Sheet_1.ZIP › Code,data and results/Figures and Tables/修改样本空间范围.doc]

	(1)	
VARIABLES	y	
		
did	1.025***	
	(4.941)	
Size	-3.229***	
	(-4.842)	
GDP	-0.932***	
	(-2.772)	
Indus	-0.051***	
	(-3.456)	
Envir	0.001	
	(0.257)	
Educa	0.012	
	(0.125)	
Open	0.008***	
	(15.092)	
		
Observations	3,407	
R-squared	0.894	
t-statistics in parentheses
*** p<0.01, ** p<0.05, * p<0.1
