# Supplementary material for: The impact of green low-carbon development on public health: a quasi-natural experimental study of low-carbon pilot cities in China
Source: Front Public Health. 2024 Oct 8;12:1470592. doi: 10.3389/fpubh.2024.1470592 (PMC11493735; doi:10.3389/fpubh.2024.1470592)
Supplement: Supplementary file 2 [file Data_Sheet_1.ZIP › Code,data and results/Figures and Tables/基准回归.doc]

	(1)	(2)	
VARIABLES	y	y	
			
did	1.467***	1.305***	
	(5.912)	(5.902)	
Size		-4.788***	
		(-7.229)	
GDP		-1.142***	
		(-3.182)	
Indus		-0.066***	
		(-4.183)	
Envir		0.001	
		(0.275)	
Educa		-0.048	
		(-0.449)	
Open		0.007***	
		(13.864)	
			
Observations	3,466	3,463	
R-squared	0.873	0.900	
t-statistics in parentheses
*** p<0.01, ** p<0.05, * p<0.1
