# Supplementary material for: The impact of green low-carbon development on public health: a quasi-natural experimental study of low-carbon pilot cities in China
Source: Front Public Health. 2024 Oct 8;12:1470592. doi: 10.3389/fpubh.2024.1470592 (PMC11493735; doi:10.3389/fpubh.2024.1470592)
Supplement: Supplementary file 2 [file Data_Sheet_1.ZIP › Code,data and results/Figures and Tables/增加控制变量.doc]

	(1)	
VARIABLES	y	
		
did	1.274***	
	(5.775)	
Size	-5.251***	
	(-7.848)	
GDP	-1.097***	
	(-3.064)	
Indus	-0.066***	
	(-4.230)	
Envir	0.002	
	(0.415)	
Educa	-0.035	
	(-0.326)	
Open	0.007***	
	(12.644)	
Density	0.005***	
	(4.344)	
		
Observations	3,463	
R-squared	0.901	
t-statistics in parentheses
*** p<0.01, ** p<0.05, * p<0.1
