# Supplementary material for: The impact of green low-carbon development on public health: a quasi-natural experimental study of low-carbon pilot cities in China
Source: Front Public Health. 2024 Oct 8;12:1470592. doi: 10.3389/fpubh.2024.1470592 (PMC11493735; doi:10.3389/fpubh.2024.1470592)
Supplement: Supplementary file 2 [file Data_Sheet_1.ZIP › Code,data and results/Figures and Tables/平行趋势检验.doc]

	(1)	
VARIABLES	y	
		
pre7	0.181	
	(0.260)	
pre6	0.798	
	(1.147)	
pre4	-0.467	
	(-0.888)	
pre3	0.040	
	(0.106)	
pre2	0.578	
	(1.510)	
pre1	0.505	
	(1.304)	
current	0.444	
	(1.134)	
post1	0.711*	
	(1.834)	
post2	0.749*	
	(1.945)	
post3	0.998**	
	(2.510)	
post4	1.585***	
	(3.285)	
post5	1.263***	
	(2.669)	
post6	1.510***	
	(3.246)	
post7	1.331***	
	(2.886)	
Size	-4.559***	
	(-6.845)	
GDP	-1.239***	
	(-3.396)	
Indus	-0.059***	
	(-3.700)	
Envir	0.001	
	(0.146)	
Educa	-0.049	
	(-0.453)	
Open	0.007***	
	(14.207)	
		
Observations	3,463	
R-squared	0.900	
t-statistics in parentheses
*** p<0.01, ** p<0.05, * p<0.1
