# Supplementary material for: The impact of green low-carbon development on public health: a quasi-natural experimental study of low-carbon pilot cities in China
Source: Front Public Health. 2024 Oct 8;12:1470592. doi: 10.3389/fpubh.2024.1470592 (PMC11493735; doi:10.3389/fpubh.2024.1470592)
Supplement: Supplementary file 2 [file Data_Sheet_1.ZIP › Code,data and results/Figures and Tables/异质性.doc]

	(1)	(2)	(3)	(4)	(5)	(6)	(7)	(8)	
VARIABLES	Phealth	Phealth	Phealth	Phealth	Phealth	Phealth	Phealth	Phealth	
									
did	1.469***	0.178	0.782**	0.121	2.349***	-0.322	1.543***	0.219	
	(0.541)	(0.199)	(0.375)	(0.271)	(0.294)	(0.313)	(0.317)	(0.367)	
Size	-14.418***	-4.215***	-5.981***	-4.819***	-7.858***	-4.676***	-9.600***	-1.927*	
	(1.546)	(0.631)	(1.555)	(1.093)	(1.005)	(1.297)	(1.244)	(1.060)	
GDP	3.231***	-0.966***	-0.228	-1.755***	-0.666	-1.063**	-0.852	-0.477	
	(1.054)	(0.317)	(0.623)	(0.417)	(0.550)	(0.440)	(0.564)	(0.473)	
Indus	-0.364***	-0.022	-0.159***	0.001	-0.036	-0.046**	-0.051**	-0.069***	
	(0.067)	(0.013)	(0.032)	(0.017)	(0.027)	(0.019)	(0.024)	(0.023)	
Envir	-0.001	0.013	0.039***	-0.004	0.003	0.000	0.008	0.019	
	(0.005)	(0.009)	(0.010)	(0.005)	(0.005)	(0.011)	(0.006)	(0.014)	
Educa	0.294	0.042	-0.450**	-0.059	0.370**	0.001	-0.122	0.195	
	(0.419)	(0.092)	(0.198)	(0.126)	(0.175)	(0.126)	(0.157)	(0.139)	
Open	0.006***	0.007***	0.006***	-0.001	0.004***	0.007***	0.005***	0.009***	
	(0.001)	(0.000)	(0.001)	(0.001)	(0.001)	(0.001)	(0.001)	(0.001)	
									
Observations	316	3,091	1,725	1,695	1,720	1,720	1,721	1,706	
R-squared	0.956	0.880	0.896	0.951	0.935	0.883	0.920	0.896	
Standard errors in parentheses
*** p<0.01, ** p<0.05, * p<0.1
