# Supplementary material for: The impact of green low-carbon development on public health: a quasi-natural experimental study of low-carbon pilot cities in China
Source: Front Public Health. 2024 Oct 8;12:1470592. doi: 10.3389/fpubh.2024.1470592 (PMC11493735; doi:10.3389/fpubh.2024.1470592)
Supplement: Supplementary file 2 [file Data_Sheet_1.ZIP › Code,data and results/Figures and Tables/描述性统计.rtf]

Variable	N	Mean	SD	Min	p50	Max	
Phealth	3466	16.36	9.502	0.443	15.73	90.44	
did	3466	0.244	0.430	0	0	1	
Size	3466	5.886	0.715	-1.514	5.935	8.138	
GDP	3466	10.51	0.696	4.595	10.52	13.06	
Indus	3466	47.30	11.35	10.68	47.77	90.97	
Envir	3466	39.52	13.66	0.360	40.32	386.6	
Educa	3463	4.698	1.153	-0.211	4.670	8.570	
Open	3466	42.83	174.9	0.0640	18.22	8234	
