# Supplementary material for: The impact of green low-carbon development on public health: a quasi-natural experimental study of low-carbon pilot cities in China
Source: Front Public Health. 2024 Oct 8;12:1470592. doi: 10.3389/fpubh.2024.1470592 (PMC11493735; doi:10.3389/fpubh.2024.1470592)
Supplement: Supplementary file 2 [file Data_Sheet_1.ZIP › Code,data and results/Figures and Tables/稳健性检验合集.docx]

|  | (1) | (1) | (1) | (1) |
| --- | --- | --- | --- | --- |
| VARIABLES | y |  |  |  |
|  |  |  |  |  |
| did | 0.588** | 1.274*** | 1.108*** | 1.025*** |
|  | (2.207) | (5.775) | (3.797) | (4.941) |
| Size | -3.774*** | -5.251*** | -5.330*** | -3.229*** |
|  | (-3.097) | (-7.848) | (-4.800) | (-4.842) |
| GDP | -1.048*** | -1.097*** | -0.862* | -0.932*** |
|  | (-2.706) | (-3.064) | (-1.772) | (-2.772) |
| Indus | -0.063*** | -0.066*** | -0.116*** | -0.051*** |
|  | (-3.167) | (-4.230) | (-5.560) | (-3.456) |
| Envir | 0.030** | 0.002 | 0.002 | 0.001 |
|  | (2.401) | (0.415) | (0.356) | (0.257) |
| Educa | -0.132 | -0.035 | -0.183 | 0.012 |
|  | (-0.910) | (-0.326) | (-1.373) | (0.125) |
| Open | 0.004*** | 0.007*** | 0.007*** | 0.008*** |
|  | (3.983) | (12.644) | (8.107) | (15.092) |
| Density |  | 0.005*** |  |  |
|  |  | (4.344) |  |  |
| Observations | 3,007 | 3,463 | 2,560 | 3,407 |
| R-squared | 0.870 | 0.901 | 0.905 | 0.894 |
